# Exome sequencing reveals a rare damaging variant in *GRIN2C* in familial late-onset Alzheimer's disease

**DOI:** 10.1186/s13195-024-01661-y

**Published:** 2025-01-14

**Authors:** Elisa Rubino, Maria Italia, Elisa Giorgio, Silvia Boschi, Paola Dimartino, Tommaso Pippucci, Fausto Roveta, Clara Maria Cambria, Gabriella Elia, Andrea Marcinnò, Salvatore Gallone, Ekaterina Rogaeva, Flavia Antonucci, Alfredo Brusco, Fabrizio Gardoni, Innocenzo Rainero

**Affiliations:** 1 Department of Neuroscience “Rita Levi Montalcini”, University of Turin, Via Cherasco 15, Turin, 10126 Italy; 2 Center for Alzheimer’s Disease and Related Dementias, Department of Neuroscience and Mental Health, AOU Città della Salute e della Scienza di Torino, University Hospital, Via Cherasco 15, Turin, 10126 Italy; 3 Department of Pharmacological and Biomolecular Sciences, University of Milan, Via Balzaretti 9, Milan, 20133 Italy; 4 Department of Molecular Medicine, University of Pavia, Via Forlanini 6, Pavia, 27100 Italy; 5 Neurogenetics Research Center, IRCCS Mondino Foundation, Pavia, 27100 Italy; 6 Medical Genetics Unit, IRCCS Azienda Ospedaliero-Universitaria, Via Albertoni 15, Bologna, 40138 Italy; 7 Department of Medical Biotechnology and Translational Medicine (BIOMETRA), University of Milan, Via Festa del Perdono 7, Milan, 20122 Italy; 8 Tanz Centre for Research in Neurodegenerative Diseases, University of Toronto, King’s College Circle 1, Toronto, ON M5S1A8 Canada; 9 Institute of Neuroscience, IN-CNR, Via Raoul Follereau 3, Vedano al Lambro, 20854 Italy; 10 Medical Genetics Unit, Città della Salute e della Scienza di Torino, University Hospital, Via Santena 19, Turin, 10126 Italy; 11 Molecular Biotechnology Center Guido Tarone, University of Turin, Piazza Nizza 44B, Turin, 10126 Italy

**Keywords:** Alzheimer’s disease, NMDAR, GRIN2C, GluN2C, 14-3-3

## Abstract

**Background:**

Alzheimer's disease (AD) is a progressive neurodegenerative disorder with both genetic and environmental factors contributing to its pathogenesis. While early-onset AD has well-established genetic determinants, the genetic basis for late-onset AD remains less clear. This study investigates a large Italian family with late-onset autosomal dominant AD, identifying a novel rare missense variant in *GRIN2C* gene associated with the disease, and evaluates the functional impact of this variant.

**Methods:**

Affected and unaffected members from a Northern Italian family were included. Genomic DNA from family members was extracted and initially screened for pathogenic mutations in *APP*, *PSEN1*, and *PSEN2*, and screened for 77 genes associated with neurodegenerative conditions using NeuroX array assay. Exome sequencing was performed on three affected individuals and two healthy relatives. Bioinformatics analyses were conducted. Functional analysis was performed using primary neuronal cultures, and the impact of the variant was assessed through immunocytochemistry and electrophysiology.

**Results:**

Pathogenic variants were not identified in *APP*, *PSEN1*, or *PSEN2*, nor in the 77 genes in NeuroX array assay. Exome Sequencing revealed the c.3215C > T p.(A1072V) variant in *GRIN2C* gene (NM 000835.6), encoding for the glutamate ionotropic receptor N-methyl-D-aspartate receptor (NMDA) type subunit 2C (GluN2C). This variant segregated in 6 available AD patients in the family and was absent in 9 healthy relatives. Primary rat hippocampal neurons overexpressing GluN2C^A1072V^ showed an increase in NMDAR-induced currents, suggesting altered glutamatergic transmission. Surface expression assays demonstrated an elevated surface/total ratio of the mutant GluN2C, correlating with the increased NMDAR current. Additionally, immunocytochemistry revealed in neurons expressing the mutant variant a reduced colocalization between the GluN2C subunit and 14-3-3 proteins, which are known to facilitate membrane trafficking of NMDARs.

**Discussion:**

We identified a rare missense variant in *GRIN2C* associated with late-onset autosomal dominant Alzheimer's disease. These findings highlight the role of GluN2C-containing NMDARs in glutamatergic signaling and their potential contribution to AD pathogenesis.

**Supplementary Information:**

The online version contains supplementary material available at 10.1186/s13195-024-01661-y.

## Introduction

Alzheimer’s disease (AD) is the most common type of neurodegenerative dementia, accounting for 50%–75% of dementia cases [1]. Symptoms are commonly divided into two categories based on the age at which symptoms begin: early-onset Alzheimer’s disease (before 65 years of age) or late-onset Alzheimer’s disease (after 65 years of age). The main clinical feature of Alzheimer’s disease is the progressive impairment of episodic memory, particularly in the ability to form new memories. As the disease progresses, other cognitive functions, such as visuospatial abilities, language skills, and executive function, also become affected. In addition to cognitive decline, individuals with Alzheimer's disease may experience neuropsychiatric symptoms, including delusions, anxiety, aggressive behavior, and wandering. The disease leads to complete dependence on others for daily activities and ultimately death [2]. This memory-focused phenotype is commonly observed in most patients with late-onset Alzheimer’s disease and a significant proportion of patients with early-onset disease [3].

Alzheimer's disease has a heritability estimate of 60–80% [4]. Rare genetic variants in the *APP* and *PSEN1* and *PSEN2* genes have been reported as pathogenic causes of early-onset Alzheimer’s disease, but they account for less than 1% of all cases [5]. Late-onset Alzheimer’s disease is the most common form of the disease, and in recent years, large-scale genome-wide association studies (GWASs) and exome/genome sequencing studies have identified more than 75 well-confirmed genetic loci that contribute to the risk of developing late-onset Alzheimer’s disease [6, 7]. These studies have captured approximately 50% of the heritability of late-onset Alzheimer’s disease, and the *APOE* ε4 allele is the major genetic risk factor for sporadic cases [8, 9]. However, a significant portion of the heritability remains unexplained, suggesting that other genetic loci may be involved in the development of the disease. It is believed that this “missing heritability” may be attributed to rare susceptibility genes that are not easily detectable via genome-wide association studies [10].

The role of the neurotransmitter glutamate and its receptors in the development of Alzheimer’s disease is still being investigated [11]. Glutamate is the primary excitatory neurotransmitter in the central nervous system and plays a crucial role in various brain functions, particularly in cortical and hippocampal regions [12]. The transmission of excitatory signals through the N-methyl-D-aspartate receptor (NMDAR) is essential for synaptic plasticity and neuronal survival [13]. However, excessive NMDAR activity can lead to excitotoxicity and contribute to neurodegeneration [14, 15]. This effect is primarily mediated by the activation of extrasynaptic NMDARs and can be counteracted by memantine, a medication currently used to treat Alzheimer's disease [16]. Notably, dysfunction in glutamatergic signaling occurs early in the progression of Alzheimer’s disease, even before the onset of cognitive decline [17]. Interestingly, proteins such as 14-3-3 play a dynamic role in regulating NMDAR trafficking, targeting the receptor to the cell surface to facilitate glutamatergic neurotransmission [18]. However, despite these findings, there is currently no clear genetic evidence supporting the involvement of glutamatergic neurotransmission in Alzheimer’s disease.

In this study, we examined a large Italian family, and we report for the first time a rare missense variant in *GRIN2C* associated with late-onset autosomal dominant Alzheimer’s disease. Furthermore, we provide data supporting a role for Glun2C-containing NMDARs and 14-3-3 proteins in Alzheimer’s disease pathogenesis.

## Methods

### Participants

This study focused on a large Italian family from the Northern region, where late-onset Alzheimer’s disease was observed. The proband sought medical assistance at the Memory Clinic, AOU Città della Salute e della Scienza di Torino, University Hospital, Turin, Italy in 2008. The patient’s caregiver reported a family history of Alzheimer’s disease, prompting a thorough investigation of all living members of the family tree. The patients in the family met the criteria set by the National Institute on Aging and Alzheimer’s Association (NIA-AA) for a probable diagnosis of Alzheimer’s disease [19, 20]. The study was conducted in accordance with the Declaration of Helsinki, and all participants provided written informed consent for the study. Ethical approval was obtained from the local Committee of the Città della Salute e della Scienza di Torino (protocol 0114576).

### Genetics analyses

Blood genomic DNA was obtained from the participants using a QIAamp DNA Mini Kit (Qiagen, Mannheim, Germany). Initially, the proband underwent screening for pathogenic mutations in *APP*, *PSEN1*, and *PSEN2* by Sanger sequencing. APP duplication was investigated by quantitative multiplex PCR of short fluorescent fragments. In 2010, the proband was then genotyped on the Illumina NeuroX array at the Clinical Genomics Centre in Toronto, Canada. This array includes the Exome BeadChip, which evaluates known pathogenic variants associated with neurodegenerative disorders and consists of approximately 24,000 variants [21].

**Fig. 1:**
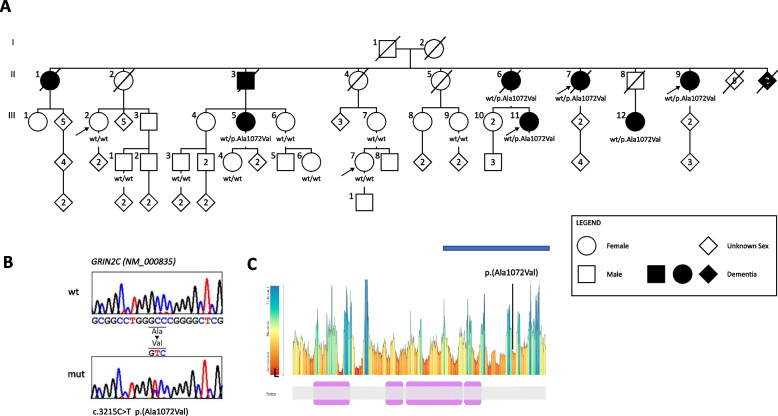
**A** Simplified pedigree of the S family. Co-segregation of *GRIN2C* p.(A1072V) with AD. To respect the privacy of the participants, the sex of some persons was masked, and the pedigree was scrambled. The arrows indicate the proband (II7) and the subjects who underwent ES (II6, II9, III2, III11, IV7). The black diamonds indicate individuals diagnosed with AD, and the pedigree shows family members with an Alzheimer's disease phenotype with segregation of the missense variant in the *GRIN2C* gene. **B** Sanger sequencing chromatogram showing the wild-type sequence and the heterozygous c.3215C > T heterozygous variant in the *GRIN2C* gene. **C** The variant tolerance of aminoacid changes in *GRIN2C* is reported using the Metadome website (https://stuart.radboudumc.nl/metadome/) [26]. The Ala1072Val change is considered “intolerant”, suggesting a pathogenic role.

Subsequently, exome sequencing (ES) was performed on three affected individuals (SF22, SF6, and SF21; Fig. 1A) and two healthy relatives (SF11 and SF27; Fig. 1A). The ES was outsourced to the Beijing Genomics Institute (BGI), which is headquartered in Shenzen, Guandong, China. Genomic DNA was used for targeted enrichment using a BGI exome kit V4 (59M, 100x). The captured libraries were then loaded onto a BGISEQ-500 platform. 200 cycle sequencing was performed for a paired-end read length of 100 bp. Bioinformatic analysis of the ES data followed well-established GATK best practices for identifying single nucleotide variants and small insertions and deletions. Briefly, reads were aligned to the hg19 reference construct built using BWA-MEM [22], followed by duplicate removal with PicardTools (http://picartools.sourceforge.net) and base quality score recalibration with GATK [23]. The alignment and coverage statistics were collected with SAMtools [24] and GATK. We generated gVCFs using HaplotypeCaller and combined them with an in-house gVCF collection to perform joint genotyping using GATK GenotypeGVCFs. Called variants were then recalibrated with GATK VariantRecalibrator and ApplyVQSR. The final VCF was annotated using VEP [25]. Sequence validation and segregation analyses were performed by Sanger sequencing using an ABI 3130XL and the ABI BigDye Terminator Sequencing Kit, V 3.1 (Life Technologies). Sequences were examined using SeqScape v2.6 software (Life Technologies).

To identify segments of identity-by-descent (IBD) that may have been inherited from a recent common ancestor, we utilized Beagle v4.1. [27]. Initially, we selected exomes from our internal database that belonged to individuals with the same ancestral background as the family under study. This included 15 unrelated individuals, as well as SF6, SF21, SF22, SF11 and SF27 individuals. The samples were obtained from the annotated multi-sample VCF file. We applied filters to retain only biallelic “PASS” variants, shared by at least two individuals of this cohort and genotyped in more than 90% of the samples. After pruning, we obtained an average rate of 500 markers per 5 Mb. Following the recommendations in the documentation, we ran Beagle using a window size of 500. Additionally, we modified the default settings for the following arguments: ‘ibd = True’, ‘ibdtrim = 15’, ‘overlap = 160’ and ‘niterations = 10.000’.

We used Merlin v.1.1.2 [28] to obtain the probability of co-segregation (linkage) between a rare allele and the disease phenotype of dementia in this family. We modeled a moderately rare trait (disease prevalence: 1%) following an autosomal dominant inheritance with penetrance 0.90 for both the heterozygous and the homozygous genotypes and performed a single-point parametric linkage analysis to calculate the corresponding LOD score. We then used R v.3.5.1 to convert the LOD score to a *p*-value with the formula: pchisq(x*(2*log(10)),df=1,lower.tail=FALSE)/2.

### Selection of candidate genes and validation of genotypes

DNA samples were processed to generate sequence reads, and a bioinformatic analysis pipeline was used to identify single nucleotide variants or small insertions and deletions. Following this bioinformatic pipeline, the resulting multi-sample VCF was postprocessed with an in-house script that allowed to retain only heterozygous and homozygous variants segregating as shared between the three affected individuals (SF22, SF6, and SF21) that corresponded to a wild-type or carrier state, respectively, in the two healthy relatives (SF11 and SF27). Only variants surviving from the following filtering steps were retained in the downstream analysis:

- 1) “PASS” variants predicted to have a consequence on the protein sequence (missense, frameshift, nonsense and splice variants);
- 2) rare variants having ≤ 0.001 max allele frequency in gnomAD v2.1.1 [29];
- 3) variants having a <  = 0.02 allele frequency in our multi-sample VCF, as we found this threshold acceptable for excluding possible sequencing artifacts or cohort-specific polymorphisms without affecting real candidate variants in the family [30];
- 4) variants having a Combined Annotation-Dependent Depletion (CADD) [31] phred score >  = 15;
- 5) Variants in genes within IBD segments.

The remaining corresponding genes were then prioritized based on their relevance to the Alzheimer’s disease phenotype using Phenolyzer (phenolyzer.wglab.org) with all default parameters, leading to the identification of a specific variant of interest. We also used Metadome, a tool for functional genomics, to identify critical protein regions where pathogenic variants are less likely to be tolerated [26].

Finally, to validate the results, 15 family members of interest were Sanger sequenced. This included 6 individuals with dementia and 9 healthy individuals.

### Cell cultures

Primary neuronal cultures from rat hippocampi at embryonic days 18–19 (E18–E19) were prepared following a previously reported method [32]. On day 10 in vitro (DIV10), neurons were transfected with calcium-phosphate method. The neurons were then used for various experiments on DIV14-15.

### Plasmids

Hippocampal neurons were transfected with pRP[Exp]-Puro- CAG>EGFP/hGRIN2C[NM_000835.6], pRP[Exp]-Puro-CAG>EGFP/hGRIN2C [NM_000835.6]* and GFP (kindly provided by Dr M. Passafaro). In all experiments, EGFP-GRIN2C plasmids were cotransfected with HA-GluN1 to generate functional NMDARs.

### Immunocytochemistry

For total staining and colocalization assays, the transfected hippocampal neurons were fixed for 10 min at room temperature (RT) in 4% paraformaldehyde (PFA) and 4% sucrose in Dulbecco’s phosphate-buffered saline (PBS). The coverslips were then washed with PBS and permeabilized with 0.1% Triton X-100 in PBS for 15 min at RT. After that, they were blocked for 45 min at RT with a solution of 5% bovine serum albumin (BSA) in PBS. The cells were then incubated with primary antibodies in 5% BSA-PBS overnight at 4°C in a humidified chamber. After being washed with PBS, the cells were incubated with fluorophore-conjugated secondary antibodies in 5% BSA-PBS for 1h at RT in a humidified chamber protected from light. After another round of washing with PBS, the cells were mounted onto glass slides using Fluoroshield mounting medium (Sigma‒Aldrich). For the surface staining assays, the cells were not permeabilized and were labelled with primary antibody for extracellular epitopes overnight at 4°C. The coverslips were then washed in PBS, and a secondary antibody conjugated to Alexa Fluor dye was used. Importantly, in all the experiments, we labelled both GluN2C^A1072V^ and GluN2C^WT^ with an antibody against the EGFP tag to which GluN2C was fused. This strategy allowed us to distinguish the specific contribution of the overexpressed GluN2C subunits from that of endogenous GluN2C. Additionally, to ensure proper delivery of the GluN2C subunit to dendrites, we cotransfected cells with a plasmid encoding the GluN1 subunit.

### Confocal imaging

Confocal Images was performed using an inverted LSM900 confocal microscope (Zeiss, Ginner, Germany) with a 63X objective. The acquired images were analyzed using ImageJ software. For colocalization analysis, the JACoP plugin was used. Cells for quantification were randomly selected from different coverslips from independent experiments, and images were acquired using consistent settings and laser power.

### Electrophysiology

The cultures were immersed in an external solution called Krebs’-Ringer’s-HEPES (KRH), which contained the following concentrations: 125 mM NaCl, 5 mM KCl, 1.2 mM KH_2_PO_4_, 2 mM CaCl_2_, 6 mM glucose, 25 mM HEPES–NaOH (pH 7.4), 1 mM TTX, 1 mM strychnine, 20 mM bicuculline, 20 mM CNQX and 10 mM glycine. Whole-cell voltage clamp recordings were performed using 14-day-old neurons that had been previously transfected with the *GRIN2C* p.A1072V and *GRIN2C* WT constructs. An Axopatch 200B amplifier and pClamp-10 software (Axon Instruments) were used, following the methods described in Pizzamiglio et al. [33, 34]. Recording pipettes were pulled from glass capillaries (World Precision Instrument) using a two-stage puller (Narishige) and had tip resistances of 3–5 MΩ when filled with the intracellular solution (130 mM Cs-gluconate, 8 mM CsCl, 2 mM NaCl, 10 mM HEPES, 4 mM EGTA, 4 mM MgATP, 0.3 mM GTP, pH 7.3). The recorded traces were analyzed using Clapfit-pClamp 10 software after selecting an appropriate threshold. NMDA whole-cell total currents were induced by applying a prolonged puff of saturating NMDA concentrations (400 µM) adjacent to the recorded neuron for a duration of 6 s, as reported in Xi et al. [35]. The holding potential throughout the experiment was − 60 mV. In the experiment involving QNZ46, the recording solution also contained QNZ46 at a concentration of 10 mM and the whole-cell recordings were repeated following the same protocol as described above. Each peak current recorded in *GRIN2C* A1072V (or *GRIN2C* WT) overexpressing neurons in the presence of QNZ46 was divided by the mean peak current recorded in *GRIN2C* A1072V (or *GRIN2C* WT)-overexpressing neurons without QNZ46, and the result is presented as a percentage.

### Antibodies and other reagents

For immunocytochemistry (ICC), the following primary antibodies were used: mouse anti-GFP (1:300, clone B38/86, Neuromab, #75–132) and rabbit anti-14-3-3 (1:125, Invitrogen, #51–0700). The following secondary antibodies were used: goat anti-mouse Alexa Fluor 488 (#411029, Life Technologies), goat anti-mouse Alexa Fluor 555 (#A21424, Life Technologies), and goat anti-rabbit Alexa Fluor 647 (#A21245, Life Technologies). The GluN2C antagonist QNZ46 was purchased from Tocris (#4801).

## Results

### Clinical findings

We investigated a large four-generation pedigree suggestive of an autosomal dominant pattern of inheritance for Alzheimer’s disease (Fig. 1A). The proband’s parents and grandparents were of Italian origin, specifically from Northern Italy. The proband was an 80-year-old right-handed woman who completed 5 years of education, admitted to the Memory Clinic of the Department of Neuroscience and Mental Health of the AOU Città della Salute e della Scienza University Hospital in 2008. She presented with mild cognitive impairment, including deficits in short-term memory, spatial–temporal disorientation, and apathy, which began at the age of 78 years. She was partially capable of performing household tasks and had moderate impairment in financial management. At the age of 72 years, she was diagnosed with major depression and treated with selective serotonin reuptake inhibitors. A strong family history of dementia was reported. Neuropsychological evaluation revealed moderate amnestic cognitive impairment with a Mini-Mental State Examination (MMSE) score of 19 out of 30. Neurological examination did not reveal any abnormalities, such as myoclonus, cerebellar signs, spastic paraparesis, or extrapyramidal signs. Brain MRI revealed bilateral frontal and parietal atrophy. A single photon emission computed tomography (SPECT) scan showed mild symmetrical decreased perfusion in the posterior parietal lobes, consistent with Alzheimer’s disease in 2008. Lumbar puncture revealed reduced Aβ42 values and increased phosphorylated tau-181 levels. Subsequently, a diagnosis of Alzheimer’s disease was given according to established criteria at the time of evaluation [19]. A posteriori, a diagnosis of AD with positive biomarkers was also made [20]. The proband carried the *APOE* ε3ε3 genotype. Both siblings were diagnosed with late-onset Alzheimer’s disease in their mid-seventies at our Memory Clinic, and one of the sisters also underwent CSF lumbar puncture, which confirmed AD diagnosis. Additionally, other five members of the pedigree have received a clinical diagnosis of late-onset AD. Eight siblings remained free from dementia until death.

### Genetic findings in family members

In 2009, no pathogenic variants were identified in the *APP*, *PSEN1*, and *PSEN2* genes in the three living patients with Alzheimer's disease, and these negative findings were also confirmed in ES. Furthermore, the NeuroX array analysis ruled out known variants associated with 77 genes associated with neurodegenerative disorders [21]. More recently, exome sequencing was conducted on five subjects, including three affected patients with AD and two healthy subjects. The statistics for ES are reported in Supplementary Table 1. Starting from the joint-genotyped, multisample VCF, we obtained 1199 variants (766 heterozygous and 433 homozygous, respectively; Supplementary Table 1). Subsequently, after each filtering steps we obtained:

- 663 “PASS” variants (435 heterozygous and 228 homozygous; Supplementary Table 2);
- 22 heterozygous rare variants in gnomAD v2.1.1 and the in-house cohort (Supplementary Table 2);
- 16 heterozygous variants with CADD phred score >  = 15 (Supplementary Table 3).

Finally, 8 of the so filtered variants were located within IBD segments (Supplementary Table 3 and Supplementary Table 4).

Although none of the corresponding genes had a clearcut relationship with Alzheimer’s disease pathobiology, *GRIN2C* received the relatively highest score in Phenolyzer analysis (0.086) on the Phenolyzer website accessed 03/2372024, against the disease terms “Alzheimer’s disease familial” and “Alzheimer’s disease late onset” (Supplementary Fig. 1). The *GRIN2C* variant is located on chromosome 17:74,842,922 and involves a C > T change in exon 13 of the coding sequence (NM_000835.6:c.3215C > T), resulting in the substitution of an alanine with a valine at protein position 1072 (p.Ala1072Val) (Fig. 1B). The p.Ala1072Val variant is likely to impact protein function according to the CADD phred score and has an extremely low allele frequency in the general population, at 2.64e-05 according to the latest gnomAD release (v.4.0). This gnomAD v.4.0 frequency corresponds to an absolute overall count of 15 alleles, all in the heterozygous state, mostly observed in the non-Finnish European population (13/15). Although the variant position currently has a sequencing coverage warning in gnomAD v.4.0 exomes (only 20% of gnomAD individuals), the allele frequency is highly consistent across gnomAD versions (Supplementary Table 3) and gnomAD v.4.0 genomes (2.63e-05, https://gnomad.broadinstitute.org/variant/17–74842922-G-A?dataset=gnomad_r4). This frequency may be compatible with the presence of individuals at risk for a late-onset condition in an unselected reference population such as gnomAD. Unfortunately, age information is available in gnomaAD v.4.0 for only 3/15 individuals, all being < 70 years old. Linkage between such a rare allele and the dementia phenotype in this family is strongly supported with a LOD score of 2.070 (*p* = 0.001). This evidence is in agreement with the extensive IBD detected on chromosome 17 (Supplementary Table 3), where 5 of the 8 rare variants selected as candidate are located.

The gene encodes GRIN2C (or GluN2C), a 1,233-amino acid protein of approximately 134 kDa. GluN2C is one of the subunits that forms the heterotetrameric structure of NMDA receptors. The p.(Ala1072Val) is located in the cytoplasmic domain of the protein (Fig. 1C). To validate our results, the p.A1072V was Sanger sequenced in 15 family members. We found that it segregated in patients with Alzheimer’s disease in the family, but not in family members who did not show any cognitive impairment (Fig. 1A).

For *APOE* genotyping, the proband was found to carry the *APOE* ε3ε3 genotype, and two affected relatives (III5 and III12) also carried ε3ε3 genotype. Using exome sequencing data and combining rs429358 (T > C) and rs7412 (C > T), *APOE* genotypes was reconstructed for other two affected relatives (II7, III11) who carried the ε2ε4 genotype and ε3ε4 genotype, respectively.

### Electrophysiological and functional studies

**Fig. 2:**
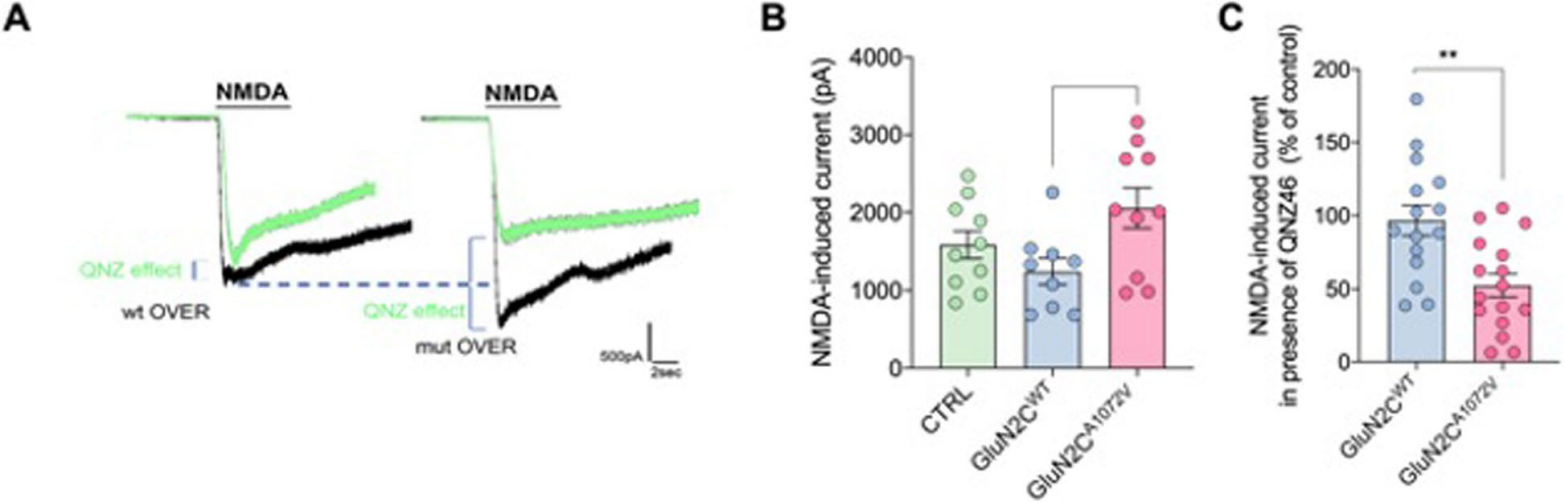
Whole cell voltage clamp recording on GluN2C^WT^ and GluN2C^A1072V^ overexpressing neurons. **A** Representative traces of NMDA whole-cell total currents induced by prolonged puff application of NMDA (400 mM) in GluN2C^WT^ (black line, left) and GluN2C^A1072V^ (black line, right) overexpressing neurons. In green, responses of GluN2C^WT^ and GluN2C^A1072V^ overexpressing neurons exposed to NMDA delivery in presence of the GluNC/D blocker QNZ46 (QNZ) in the extracellular solution. **B** Analysis of NMDA-induced responses generated in untreated, GluN2C^WT^ and GluN2C^A1072V^ cells (one-way ANOVA, Tukey's multiple comparisons test, *P* = 0.035; each dot indicates the NMDA current generated in a single neuron). **C** Analysis of NMDA responses generated in GluN2C^WT^ vs GluN2C^A1072V^ cells in the presence of QNZ and expressed as a percentage of the control, i.e., NMDA responses recorded in a solution without QNZ (two-tailed unpaired t test, *P* = 0.002). Normal distribution was checked using the D'Agostino & Pearson normality test. Bar graphs show the mean ± s.e.m

**Fig. 3:**
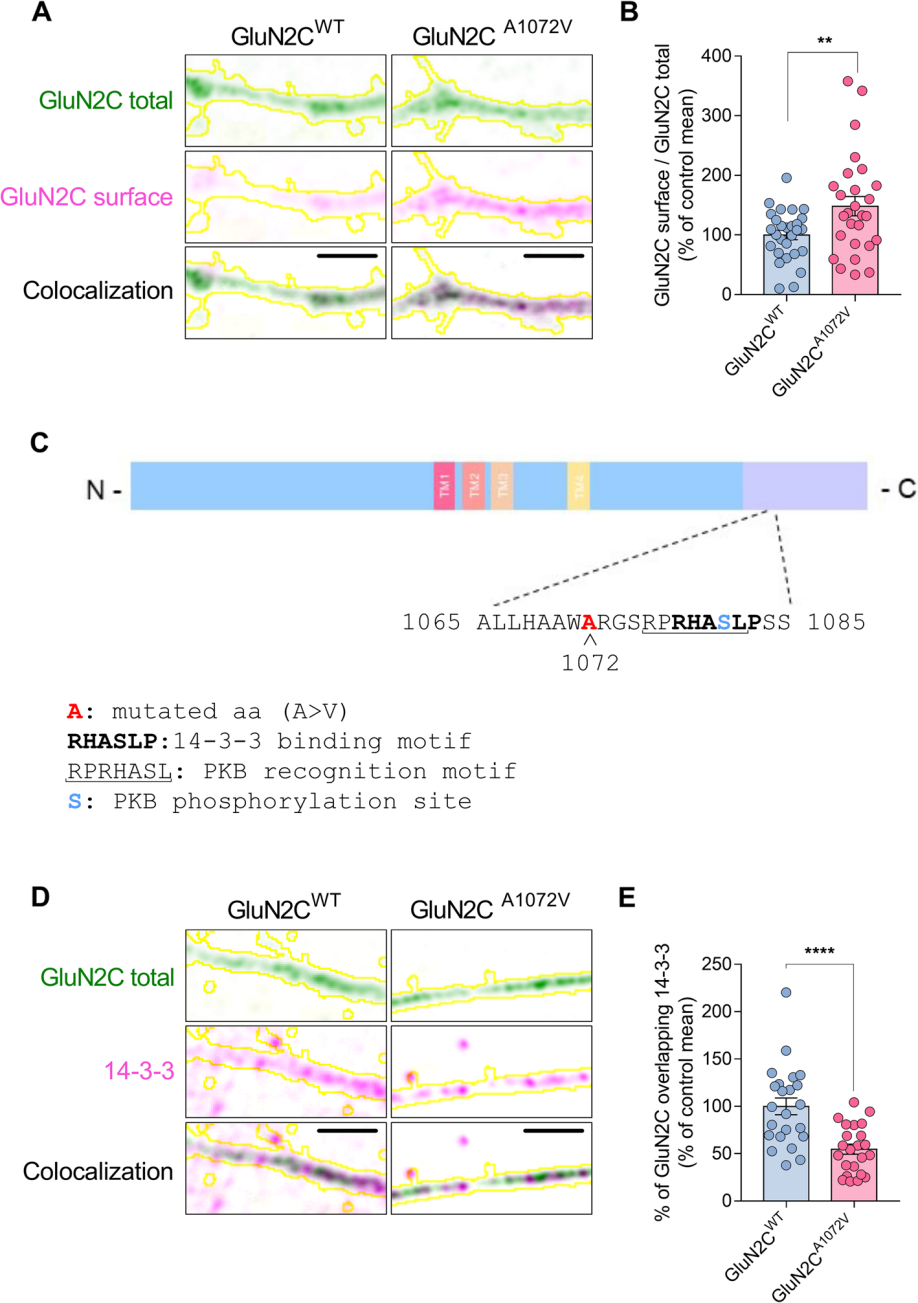
GluN2C^A1072V^ amino acid substitution in the *GRIN2C* gene promotes GluN2C surface expression and negatively affects the interaction of GluN2C with 14-3-3 scaffolding proteins. **A** Confocal images of surface (magenta) and total (green) GluN2C staining in GluN2C^WT^ (left)- or GluN2C^A1072V^ (right)-overexpressing neurons (yellow outline). Scale bar: 3 μm. **B** Bar graph representing the percentage of the control mean GluN2C surface/total ratio (two-tailed unpaired t test; t(53) = 2.693, *p* = 0.0095, *n* = 28–27/group from 4 independent experiments). **C** Schematic representation of the GluN2C protein structure. In the figure, the amino acid substitution (A1072V), the 14-3-3 binding motif, the PKB recognition motif and the PKB phosphorylation site are highlighted. **D** Confocal images of 14-3-3 (magenta) and total GluN2C (green) staining in GluN2C^WT^ (left)- or GluN2C^A1072V^ (right)-overexpressing neurons (yellow outline). Scale bar: 3 μm. **E** Bar graph representing the percentage of the control mean percentage of % of GluN2C colocalized with 14-3-3 (two-tailed unpaired t test; t(44) = 4.411, *p* < 0.0001; *n* = 23–23/group from 3 independent experiments). Bar graphs show the mean ± s.e.m. *n* = number of neurons

To evaluate the impact of GluN2C^A1072V^ change on NMDAR function, we conducted whole-cell voltage clamp recording on neurons overexpressing both GluN2C^WT^ and GluN2C^A1072V^. We elicited NMDA whole-cell total currents by applying a prolonged puff of saturating NMDA concentrations [35]. The NMDA was delivered to the recorded neuron for 6 s. As shown in Fig. 2A, B, we observed an elevated peak current mediated by NMDA in cells overexpressing GluN2C^A1072V^, suggesting that this change may result in abnormal glutamatergic transmission when NMDAR is activated. Additionally, we replicated the electrophysiological experiments in cultures where the recording solution contained the GluNC/D blocker QNZ46. We evaluated the response induced by NMDA by delivering NMDA puffs. The results shown in Fig. 2C demonstrate that QNZ46 acts more effectively in neurons overexpressing GluN2C^A1072V^ than in those expressing the wild-type protein. This finding confirms that the higher peak current generated by NMDA delivery is a direct result of the GluN2C^A1072V^ change. To investigate whether the observed increase in NMDAR currents in neurons overexpressing GluN2C^A1072V^ is due to an increase in NMDAR surface localization compared to GluN2C^WT^, we conducted a surface/total assay to evaluate the surface levels of GluN2C in both GluN2C^WT−^ or GluN2C^A1072V^-overexpressing neurons. Our findings indicated that the overall surface/total ratio of GluN2C staining along dendrites was significantly higher in neurons expressing the mutant form of the subunit (Fig. 3A, B). This result is consistent with the enhanced effect of QNZ46-mediated GluN2C inhibition in neurons overexpressing the mutant protein. Specifically, the greater the amount of GluN2C on the cell surface, the more pronounced the effect of its inhibition (Fig. 3C).

After analyzing the amino acid sequence of GluN2C, we noticed that the site of the A1072V amino acid substitution site is located near the binding motif of 14-3-3 scaffolding proteins (Fig. 3C). Notably, these proteins are known to be involved in promoting the surface expression of several membrane proteins, including the NMDAR subunit GluN2C [36]. Based on this knowledge, we hypothesized that the A1072V amino acid substitution could potentially affect the interaction between GluN2C and 14-3-3. To test this hypothesis, we used a pan 14-3-3 antibody that allowed us to detect the colocalization of GluN2C with all isoforms of 14-3-3 proteins. As shown in Fig. 3D and E, we observed a significant decrease in the colocalization of GluN2C with 14-3-3 proteins along dendrites in neurons expressing GluN2C^A1072V^ compared to those expressing GluN2C^WT^.

When GluN2C-containing NMDARs are phosphorylated at Ser1081 by PKB, they can interact with 14-3-3 proteins. This interaction facilitates their movement from the endoplasmic reticulum to the cellular membrane. Based on this knowledge, we hypothesized that the A1072V amino acid substitution could potentially affect the interaction between GluN2C and 14-3-3. To test this hypothesis, we used a pan 14-3-3 antibody that allowed us to detect the colocalization of GluN2C with all isoforms of 14-3-3 proteins. As shown in Fig. 3D and E, we observed a significant decrease in the colocalization of GluN2C with 14-3-3 proteins along dendrites in neurons expressing GluN2C^A1072V^ compared to those expressing GluN2C^WT^.

## Discussion

To our knowledge, this study was the first to identify a *GRIN2C* missense variant in late-onset Alzheimer's disease with functional consequences on NMDAR-induced currents and interaction with 14-3-3 scaffolding proteins.

Late-onset Alzheimer’s disease has a significant genetic component, with heritability estimates suggesting that genetic factors account for 60% to 80% of the disease [37]. Previous GWASs have identified more than 50 genetic loci associated with late-onset AD, primarily in individuals of European ancestry [38, 39]. However, these studies have mostly identified common variants, and it is believed that only approximately half of the genetic heritability of Alzheimer's disease has been explained thus far. To address this limitation, researchers are now employing next-generation strategies, such as exome sequencing and genome sequencing, to identify rare variants that contribute to Alzheimer’s disease. A recent exome sequencing study identified rare potentially damaging variants in the *ATP8B4* and *ABCA1* genes that are associated with AD risk [40]. This study highlights the importance of looking beyond common variants and exploring rare variants to fully understand the genetic basis of the disease. In line with these efforts, our study adds a novel gene to the growing body of evidence supporting the role of rare variants in Alzheimer's disease.

*GRIN2C* encodes the glutamate ionotropic N-Methyl-D-aspartate receptor (NMDA) type subunit 2C (also called GluN2C), and the subunits of NMDA receptors (NMDARs) are known for their distinct roles in regulating synaptic plasticity, as well as influencing processes related to learning and memory. In literature, several studies support the involvement of the neurotransmitter glutamate and its receptors in the pathogenesis of Alzheimer’s disease. Notably, NMDA receptors that contain the GluN2C subunit exhibit distinctive biophysical characteristics and exhibit unique expression patterns. Depletion of the GluN2C subunit within the prefrontal cortex of mice led to a shift in the balance between excitation and inhibition, favoring inhibition [41]. Furthermore, the absence of GluN2C activates intracellular signaling pathways that support neuronal survival, as demonstrated by heightened levels of nuclear cAMP response element-binding protein [42].

Here, we demonstrated that hippocampal neurons overexpressing the mutant form of GluN2C^A1072V^ have an increase in NMDAR-induced currents, suggesting that this mutation may enhance NMDAR function. Interestingly, a recent study revealed that the expression of the GluN2C subunit decreases under the influence of memantine, and memantine also induces alterations in the level of GRIN2C mRNA within the prefrontal cortex [43]. The authors also reported a shift in the expression levels of the GluN2C subunit, resulting in a noteworthy reduction in excitability within cortical structures, although receptors containing the GluN2C subunit are markedly less prevalent in the brain when compared to receptors harboring GluN2A and GluN2B [43].

In our study, when we examined the surface retention of the GluN2C mutant form, we found that it was more abundant than that of the wild-type form, and this finding further supports the notion that the variant alters the function of GluN2C.

Glutamate represents the foremost essential excitatory neurotransmitter within synaptic systems, accounting for approximately 40% of all synapses. Altered glutamatergic neurotransmission could represent a common factor, providing a unifying perspective to explain the neurocognitive impairments and symptomatic features observed in different neurological disorders. The relationships between the pathological hallmarks of Alzheimer’s disease, such as amyloid-beta (Aβ) deposition and Tau hyperphosphorylation, and glutamatergic transmission have mainly been studied in vitro [44, 45]. Several reports have shown that Aβ oligomers and NMDARs contribute to synaptic dysfunction and disruption in AD. The effect of Aβ on synapses is to alter glutamatergic synaptic transmission and decrease the number of surface receptors for glutamate and other synaptic components in hippocampal neurons [46]. The loss of Ca^2+^ homeostasis is believed to be connected to the early cognitive deficits observed in AD. Additionally, Tau, a major component of neurofibrillary tangles, plays a crucial role in regulating synaptic function. Excess tau in Alzheimer’s disease dendrites controls NMDA receptor activity, leading to an increase in calcium levels in dendrites that can reach damaging levels. This calcium-driven excitotoxicity can harm postsynaptic sites and result in neuronal death [47]. These studies have demonstrated that dysregulation of NMDA receptors can lead to excitotoxicity, and the current study extends this knowledge by identifying a variant in the *GRIN2C* gene, suggesting that other NMDA receptor subunits might also contribute to AD pathogenesis. In this direction, interestingly, a recent study on multivariate GWAS in Alzheimer’s disease that investigated the role of genetic variants on CSF biomarker profiles showed a genome-wide significant gene-based association with variants in *GRIN2D* and the synaptic functioning components, further supporting a role of altered synaptic function in AD [48].

Additionally, we also discovered that GluN2C^A1072V^ has increased localization at the cell surface compared to GluN2C^WT^, and this event is associated with a reduced colocalization with its known partner, 14-3-3. Overall, these results suggest an alteration of physiological mechanisms involved in 14-3-3-dependent trafficking at neuronal membranes, leading to an aberrant accumulation of the NMDA receptor subunit at the cell surface. A reasonable explanation is that, once inserted into the membrane, GluN2C rapidly dissociates from 14-3-3 (working mainly as a cargo and not as scaffolding protein), thus explaining the observed reduced colocalization between the two proteins. The mammalian 14-3-3 family comprises seven evolutionarily conserved proteins that bind several protein targets, thereby influencing cell signaling pathways. The presence of 14-3-3 proteins in cerebrospinal fluid serves as a sensitive and specific biomarker for neuronal damage associated with several neurological diseases, including Creutzfeldt‒Jakob disease, Alzheimer's disease, brain cancers, and stroke [49]. In our study, we discovered that the GluN2C^A1072V^ has a negative impact on the co-localization of GluN2C with the known partner 14-3-3, suggesting a reduced interaction with these scaffolding proteins. Therefore, the pathogenic effect of the variant may be related to this altered protein interaction. Another recent study compared crosslinking in aggregate proteins isolated from the hippocampus of Alzheimer’s disease patients with that from controls, and the authors found a total of 85 interacting proteins that were specifically associated with 14-3-3 only in the tissue from AD patients [50]. 14-3-3 proteins function as molecular chaperones, helping to prevent protein unfolding and aggregation during cellular stress. Therefore, the impaired binding between GluN2C and 14-3-3 may be relevant to the neurodegenerative process in these patients.

There are some limitations of this study that should be acknowledged. First, the diagnosis of Alzheimer’s disease in the examined pedigree has not yet been confirmed through pathological examination. However, the diagnosis was made using the NIA-AA Research Framework criteria, which have been shown to have a significant correlation with pathological confirmation in previous studies [19]. It is important to note that this study was conducted in a single family, and therefore, the observed pathogenic variant can currently only be classified as specific to this family. Further research is necessary to determine the prevalence of genetic variants in the *GRIN2C* gene in late-onset Alzheimer's disease.

In conclusion, this study presents findings on a family in which late-onset Alzheimer's disease is associated with the p.(Ala1072Val) variant in the *GRIN2C* gene. Additionally, this study provides interesting data that support the involvement of GluN2C-containing NMDARs and 14-3-3 proteins in the pathogenesis of Alzheimer's disease.

## Supplementary Information

Supplementary Material 1.

Supplementary Material 2.

## Data Availability

No datasets were generated or analysed during the current study.
