# Supplementary figures and images for: Exome sequencing reveals a rare damaging variant in GRIN2C in familial late-onset Alzheimer's disease
Source: Alzheimers Res Ther. 2025 Jan 14;17:21. doi: 10.1186/s13195-024-01661-y (PMC11730494; doi:10.1186/s13195-024-01661-y)

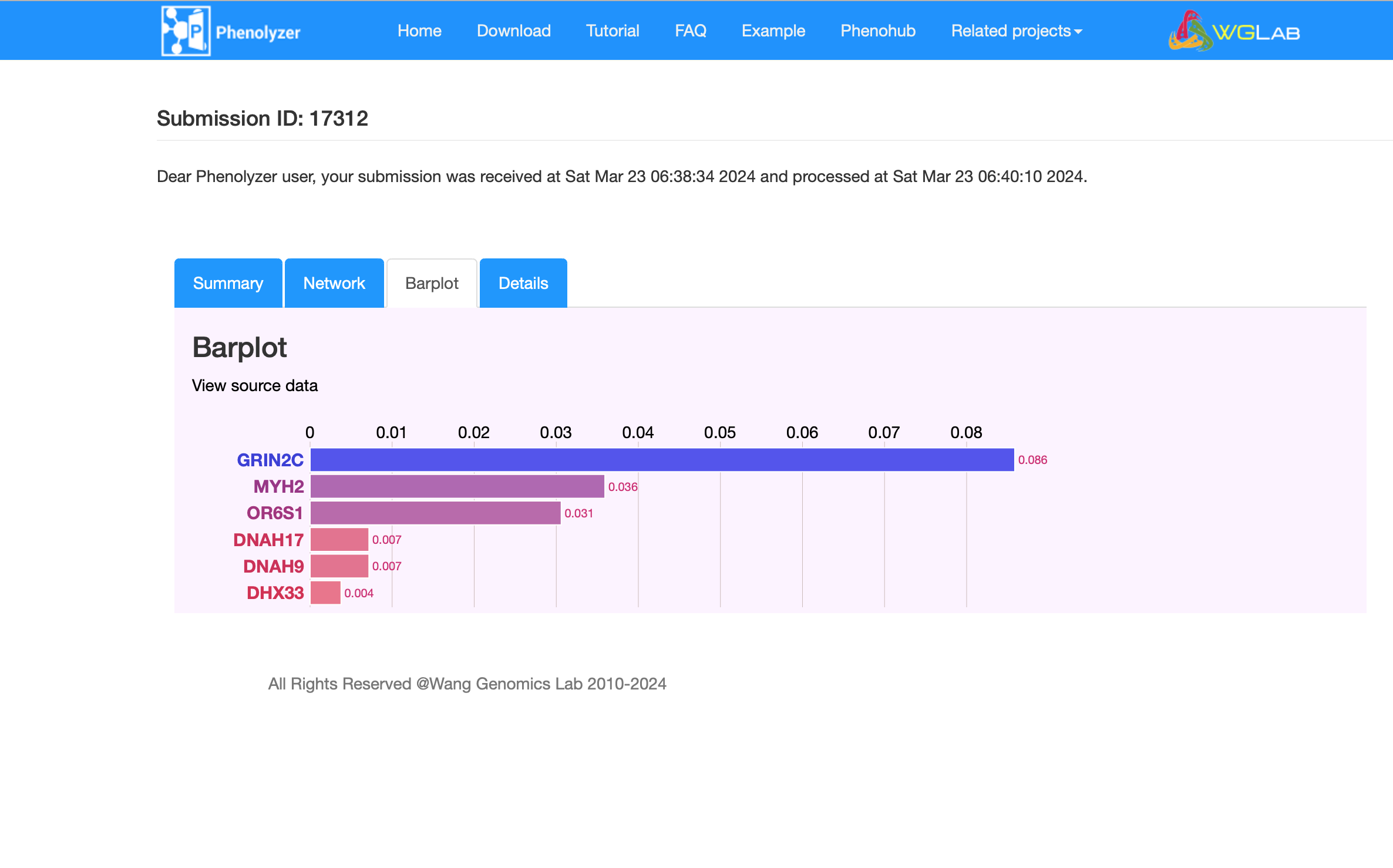

Supplement: Supplementary file 1 — Supplementary Material 1. [file 13195_2024_1661_MOESM1_ESM.png]
